# Comprehensive circular RNA profiling provides insight into colorectal cancer pathogenesis and reveals diagnostically relevant biomarkers

**DOI:** 10.1002/ctm2.70049

**Published:** 2024-10-13

**Authors:** Shujin Li, Jun Wang, Lin Qiu, Gaohui Fu, Yang Li, Qiang Su, Yiheng Zhu, Feilong Zhao, Jinglin Tian, Jinyong Huang, Yanqin Niu, Kang Kang, Deming Gou

**Affiliations:** ^1^ Shenzhen Key Laboratory of Microbial Genetic Engineering, Vascular Disease Research Center, College of Life Sciences and Oceanography, Guangdong Provincial Key Laboratory of Regional Immunity and Disease, Carson International Cancer Center, School of Medicine Shenzhen University Shenzhen China; ^2^ Department of Gastrointestinal Surgery Second Clinical Medical College of Jinan University, Shenzhen People's Hospital Shenzhen China; ^3^ College of Physics and Optoelectronic Engineering Shenzhen University Shenzhen China; ^4^ College of Medicine Shenzhen University Shenzhen China

Dear Editor

The incidence of colorectal cancer (CRC) is increasing, especially among younger populations, underscoring the necessity for a thorough examination of biomarkers.^1^, ^2^ This study explores the clinical and functional roles of circRNAs in CRC using a stage‐stratified, integrated multiomics approach. The identified circRNA panel holds promise for CRC diagnosis.^3^, ^4^

Utilising whole transcriptome sequencing on tumour, normal, and paracancer tissues from 30 CRC patients, circRNA alterations were investigated (Table S1). CircRNAs were identified with CIRI2 and DCC software (Table S2).^5^, ^6^ Increased sequencing depth correlated with a higher number of identified circRNAs (Figure S1A–D). Tumour tissues exhibited a lower abundance of circRNAs compared to normal and paracancer tissues (Figure S1 E–H), indicating changes in circRNA expression during CRC pathogenesis.^3^ To minimise variability, we focused on circRNAs detectable in at least half of the patients (Figure 1A and B), which revealed more distinct tissue‐specific differences in circRNA numbers (Figures 1C and S1I); further analysis revealed high consistency between cirRNAs identified by CIRI2 and DCC (Figure 1D–F). In subsequent analysis, only circRNAs detected by both software were considered (Figure S1J–L). Our investigation showed that most circRNAs corresponded to a single host gene and were predominantly exon‐type (Figure 1G and H). Despite the reduced number of circRNAs in tumours, their chromosomal distribution was similar to those in normal and paracancer tissues (Figure S2A and B). Annotation of the circRNAs using circBase supports the reliability of our identification process (Figure 1I).

**FIGURE 1:**
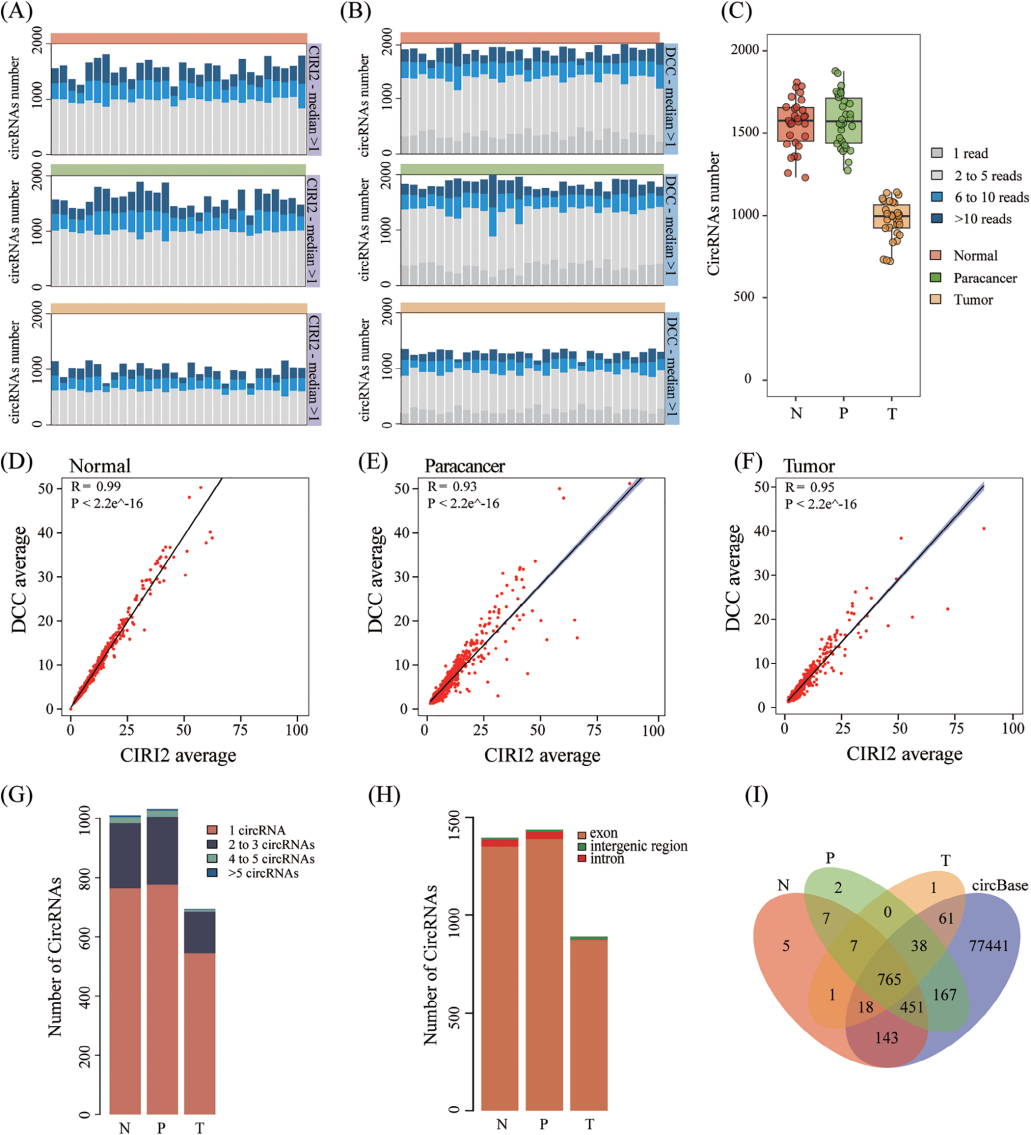
Identification and characterisation of circRNAs in CRC tissues. (A, B) The numbers of circRNAs identified in each tissue type after removing low‐quality back‐splice junction (BSJ) reads for CIRI2 (A) and DCC (B). Each bar represents a patient sample. (C) Number of circRNAs identified per patient in each tissue type after filtering out low‐quality BSJ reads. (D–F) Scatter plots showing the correlation between circRNAs identified by CIRI2 and DCC in normal (D), paracancer (E), and tumour (F) tissues. (G, H) Bar chart displaying the number of circRNAs generated from different host gene types (G) and genomic region (exon, intergenic region, and intron) (H) in each tissue sample. (I) Annotation of identified circRNAs in known databases across the three tissues types.

We further identified 67 and 71 differentially expressed circRNAs (DEcircRNAs) in tumour versus normal (N vs. T) and paracancer versus tumour (P vs. T), respectively (Figure S2C and D). Notably, no DEcircRNAs were found in the normal versus paracancer comparisons (N vs. P) (Figure S2E). Focusing on the 55 DEcircRNAs in tumour tissues (Figure 2A and Table S3), these circRNAs effectively distinguished tumour from normal or paracancer tissues in both heatmap, and principal component analysis (PCA) (Figures 2B and S2F and G), outperforming the top 50 highly expressed circRNAs. Although these circRNAs did not show specific chromosomal enrichment, they significantly overlapped with known CRC‐sensitive mutagenic segments (Figure 2C), suggesting a potential association with mRNA imbalance in these regions. Positive correlations between circRNA expression changes and their host genes comparisons support the hypothesis of host gene‐driven circRNA changes (Figures 2D and E and S2H).

**FIGURE 2:**
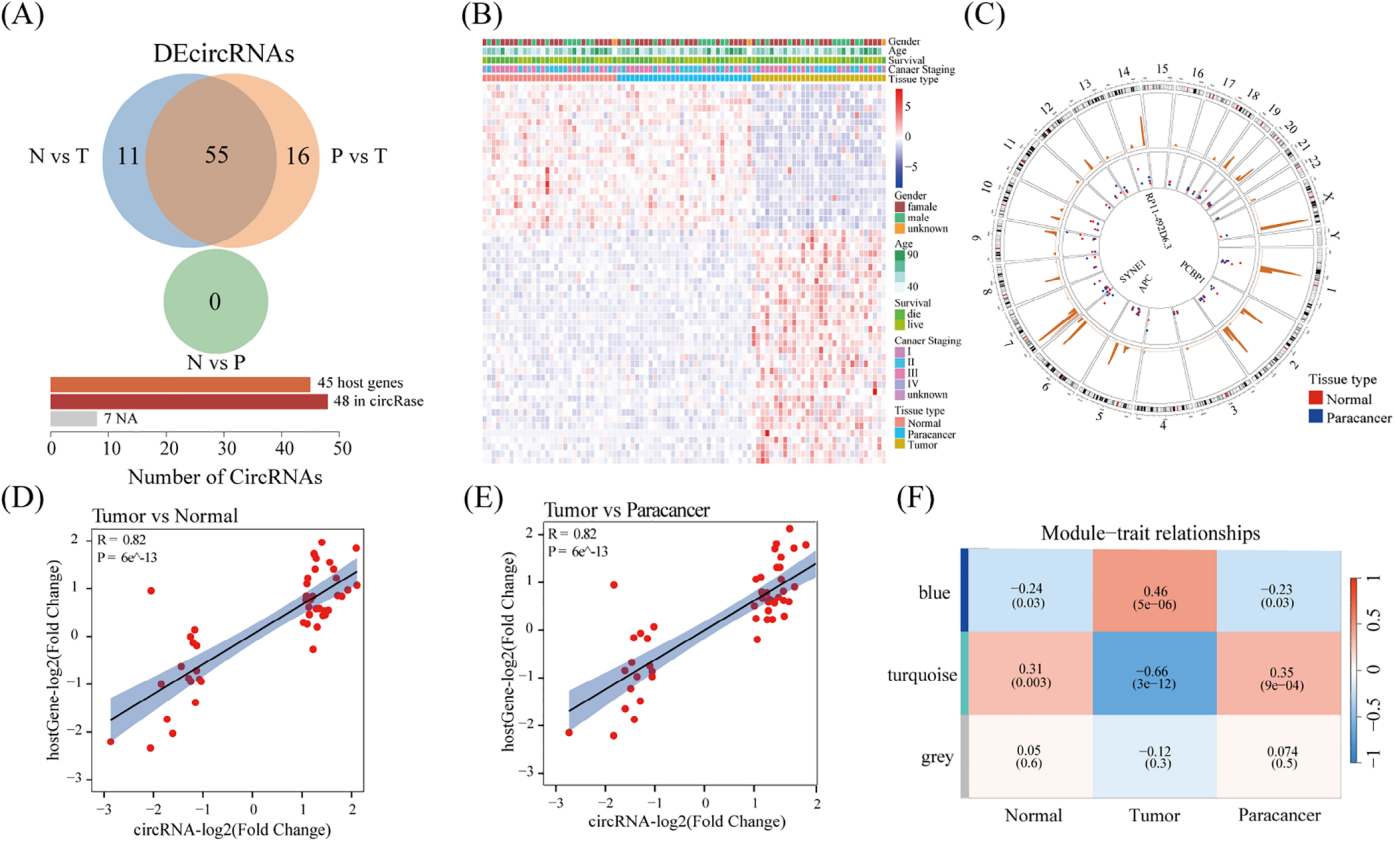
Screening of circRNAs associated with CRC. (A) Venn diagram showing circRNAs meeting specific criteria: significant difference in normal versus tumour, significant difference in paracancer versus tumour, and no significant difference in normal versus paracancer. (B) Heatmap displaying log2(TPM) values of the circRNAs across the three tissue types. (C) Chromosomal distribution of the circRNAs, regions susceptible to CRC mutations are highlighted in red. (D, E) Scatter plots depicting the correlation between log2(fold Change) of the circRNAs and their host genes in normal versus tumour (D) and paracancer versus tumour (E) comparisons. (F) Three modules were revealed by WGCNA.

Applying WGCNA to the circRNA dataset (Figure S3A–C), we identified two co‐expression modules significantly linked to CRC, which further helped to identify tumour tissue‐specific circRNAs (Figure 2F and Table S4). Investigation of the top 50 circRNAs from these modules unveiled distinct functional enrichment patterns (Figure S3D and E).

Integrating WGCNA and DEcircRNA results, we identified 36 CRC tumour‐specific circRNA (Figure S3F and G and Table S5). Subsequently, a circRNA‐miRNA‐mRNA regulatory network was constructed, incorporating miRNAs exhibiting opposite expression trends to their targeted mRNAs, among which three key regulatory axes was further validated by qRT‐PCR with ABCE1, FABP4 and RNF103 showed concordant expression pattern with hsa_circ_0001461, hsa_circ_0087960, and hsa_circ_0019223 (Figures 3A and S3H–K). GO and KEGG analysis on the targeted mRNAs revealed enrichment in cancer‐related pathways, such as Ras signalling and chemokine signalling (Figure 3B).^7^, ^8^ Gene Set Enrichment Analysis (GSEA) confirmed pathways like chemokine signalling and cytokine‐cytokine receptor interaction for upregulated genes, and pathways in cancer and metabolic pathways for downregulated genes (Figure 3C and D).

**FIGURE 3:**
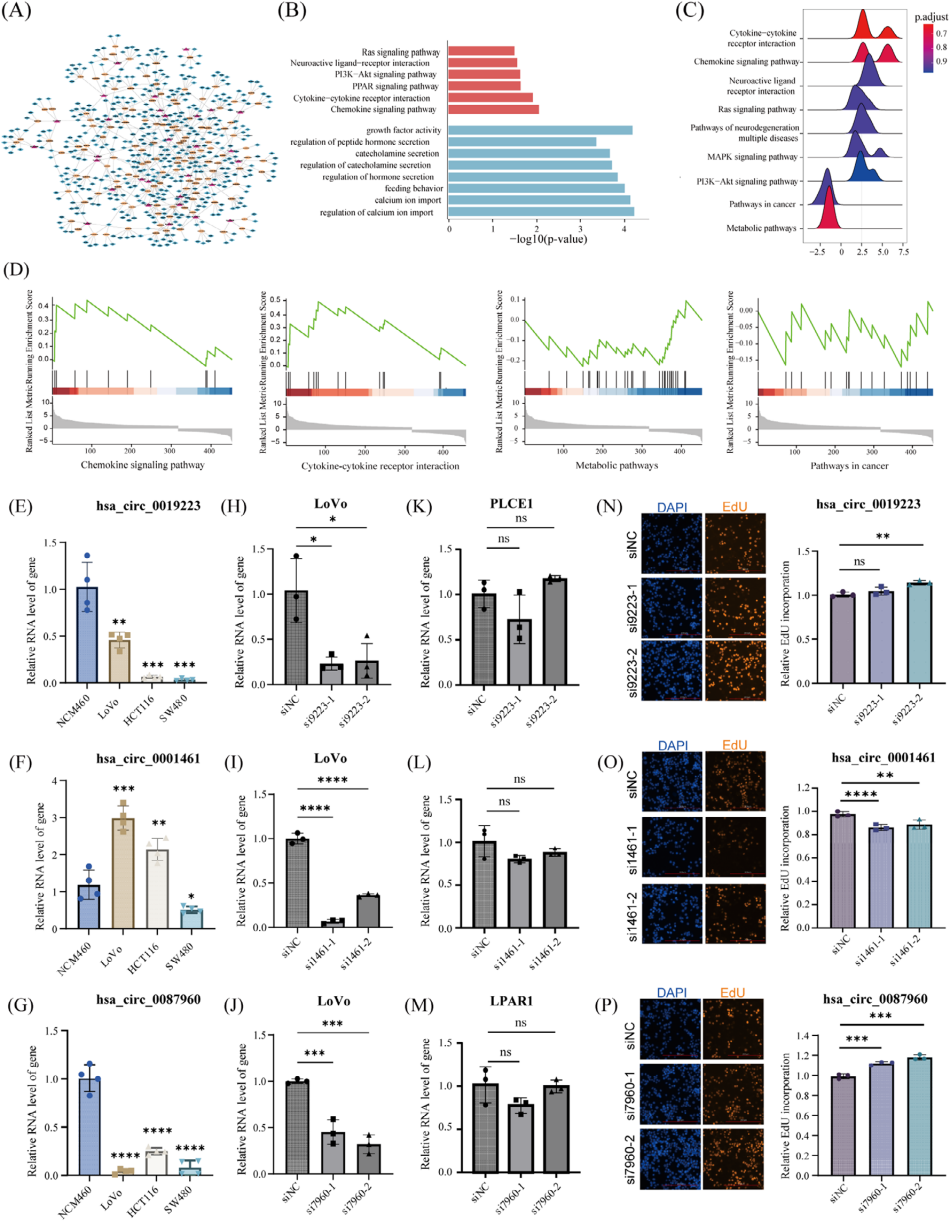
CircRNA‐miRNA‐mRNA regulatory network construction and functional validation. (A) Regulatory network representation of the predicted circRNA‐miRNA‐mRNA regulatory axes, involving 30 circRNAs, 126 miRNAs, and 457 mRNAs. (B) GO and KEGG enrichment analysis results for the targeted mRNAs associated with the identified miRNAs. (C, D) KEGG pathway analysis and GSEA revealed enrichment of 457 targeted mRNAs in multiple intriguing pathways particularly in 4 cancer‐related pathways (Chemokine signalling pathway, Cytokine‐cytokine receptor interaction, Metabolic pathways, and Pathways in cancer). (E–G) Expression profiles of hsa_circ_0019223 (E), hsa_circ_0001461 (F) and hsa_circ_0087960 (G) in CRC cell lines (LoVo, HCT116, and SW480) and the normal cell line NCM460. (H–M) qRT‐PCR analysis following siRNA transfection in LoVo cell line to assess the expression of the circRNA and their corresponding host genes: hsa_circ_0019223 (H and K), hsa_circ_0001461 (I and L) and hsa_circ_0087960 (J and M). (N–P) EdU assay showed that silencing of hsa_circ_0019223 (N) and hsa_circ_0087960 (P) led to increased cell proliferation, while silencing of hsa_circ_0001461 (O) resulted in decreased cell proliferation. Significance levels are indicated by *p < .05, **p < .01, ***p < .001, ****p < .0001. Data are presented as mean ± SEM.

To evaluate the clinical relevance of the DEcircRNAs, we assessed their expression in two independent datasets (GSE221240 and GSE235850). The log2FoldChange calculations demonstrated consistent expression patterns for most DEcircRNAs (Figure S4A). Notably, eight circRNAs exhibited exceptional diagnostic efficacy for CRC, with hsa_circ_0073244 displaying outstanding discriminatory power (AUC = 0.9717, 95% CI: 0.9398‐1.000) (Figure S4B). The expression patterns of hsa_circ_0019223, hsa_circ_0001461 and hsa_circ_0087960 were validated in three CRC cell lines, aligning with observations in CRC tumour tissues (Figure 3E–G). To explore the functional roles of these DEcircRNAs, we designed two siRNAs targeting the BSJ region of each circRNA and performed silencing assays in LoVo cells. qRT‐PCR confirmed effective silencing of the three DEcircRNAs without affecting their host genes (Figure 3H–M). Cell proliferation was subsequently assessed using EdU assay, which indicated that silencing hsa_circ_0019223 and hsa_circ0087960 led to a significant increase in cell proliferation, whereas silencing hsa_circ_0001461 resulted in a marked decrease in proliferation (Figure 3N–P).

We further stratified patients into early‐ (*n* = 14) and late‐stage (*n* = 14) groups, and identified significant changes in hsa_circ_0008039, hsa_circ_0004865 and hsa_circ_0077837 expression between groups (Figure S4C), suggesting the potential of these circRNAs as staging biomarkers.

We employed qRT‐PCR to validate the expression of the 8 identified circRNAs and their host genes. The results matched the RNA‐seq trends, suggesting similar expression patterns of DEcircRNAs and their host genes in tumour tissues (Figure S5A and B). Further sequencing confirmed the predicted BSJ sites (Figure S5A). Additional validation using 10 CRC tumour‐normal tissue pairs supported the consistent expression patterns of these circRNAs (Figure S6A). These validations enhance confidence in the circRNA expression profiles and suggest their potential as CRC biomarkers.

As the expression pattern of DEcircRNAs in this study closely resembled those of their corresponding host genes (Figures 2D–E and S5), we further analysed the single‐cell RNA sequencing dataset (GSE161277) from tumour, paracancer, and normal tissues. This analysis revealed the cellular distribution of the host genes, identifying 21 distinct cell populations across 43,851 cells from 13 samples (Figure 4A and B and Table S6). Marker genes for each cell type were visualised in a bubble plot (Figure 4C). The host genes of 11 previously identified circRNAs exhibited gradual expression changes in fibroblasts and epithelial cells.^9^ Host genes were enriched in specific cell types, such as PTPN22 and PLCE1 in CD8+ T cells, PRKAR1B in endothelial cells, and SPATA13 in Tcm+ Tfh+ Treg cells (Figures 4D and S7A). Notably, hsa_circ_0000110 (PTPN22) showed significant enrichment in plasma B cells (Figure 4E), indicating its potential as a non‐invasive diagnostic biomarker. The scRNA‐seq data also revealed dynamic changes in host gene expression during tumour progression across different cell types, suggesting potential roles of circRNAs in cellular transformation.

**FIGURE 4:**
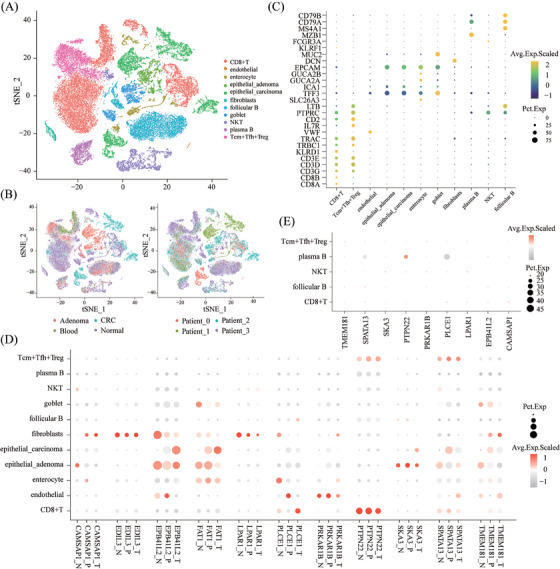
Single‐cell analysis of host gene expression of the identified circRNAs. (A) t‐SNE plot visualising single cells (*n* = 13 samples, 4 patients) coloured by distinct cell types (11 in total, listed in figure). (B) t‐SNE plots depicting cell distribution based on tissue source (left) and patient source (right). Dots represent individual cells, coloured by tissue type or patient ID. (C) Marker genes for each identified cell cluster. Circle size represents the percentage of cells expressing the gene, while colour represents the average expression within the cluster. (D) Distribution of host genes for 11 circRNAs within different cell types across three sample types. Circle size represents the percentage of cells expressing the gene, and colour represents the average expression within the cluster. (E) Distribution of host genes for 9 out of the 11 circRNAs within different cell types from plasma samples. Circle size represents the percentage of cells expressing the gene, and colour represents the average expression within the cluster.

In summary, our study pinpointed 11 circRNAs strongly associated with CRC development. These circRNAs hold promise as early diagnostic biomarkers.

## AUTHOR CONTRIBUTIONS

J.W., S.L. and D.G. designed and supervised the study. Y.L. collected and provided 90 cases of CRC tissue samples, L.Q. and G.F. performed bioinformatics analysis and functional experiment. S.L. interpreted the results and drafted the manuscript, with assistance from G.F. in multiomics analysis. J.W. and D.G. supervised and supported the work. J.W. and D.G. revised the manuscript. J.W., D.G., S.L. and L.Q. discussed data integrity and critically reviewed each part for publication. J.W. and D.G. were responsible for quality control. J.W., D.G., S.L., L.Q., G.F., Q.S., Y.Z., F.Z., J.T., J.H., Y.N. and K.K. assisted in resource collection and approved the final manuscript.

## CONFLICT OF INTEREST STATEMENT

The authors declare that no conflict of interest.

## ETHICS STATEMENT

This study was approved by the institutional review board of Shenzhen University and Shenzhen People's Hospital. Written informed consent was obtained from all patients. All procedures were in accordance with the Declaration of Helsinki.

## Supporting information



Supporting information

Supporting information

Supporting information

Supporting information

Supporting information

Supporting information

Supporting information

Supporting information

Supporting information

Supporting information

## Data Availability

Sequencing data in this study have been deposited at National Genomics Data Center (NGDC) with the accession number PRJCA019939 and are publicly available as of the date of publication. All original code has been deposited at GitHub and is publicly available as of the data of publication (https://github.com/wangjun‐bio/miRlab/tree/CRC). The scRNA‐seq data were downloaded from the Gene Expression Omnibus (GEO) database with accession number GSE161277. Two circRNA expression matrix datasets were downloaded from the GEO database with accession number GSE221240 and GSE235850.
