# Supplementary material for: Comprehensive circular RNA profiling provides insight into colorectal cancer pathogenesis and reveals diagnostically relevant biomarkers
Source: Clin Transl Med. 2024 Oct 13;14(10):e70049. doi: 10.1002/ctm2.70049 (PMC11471576; doi:10.1002/ctm2.70049)
Supplement: Supplementary file 1 — Supporting information [file CTM2-14-e70049-s007.docx]

**Supplementary Figures**


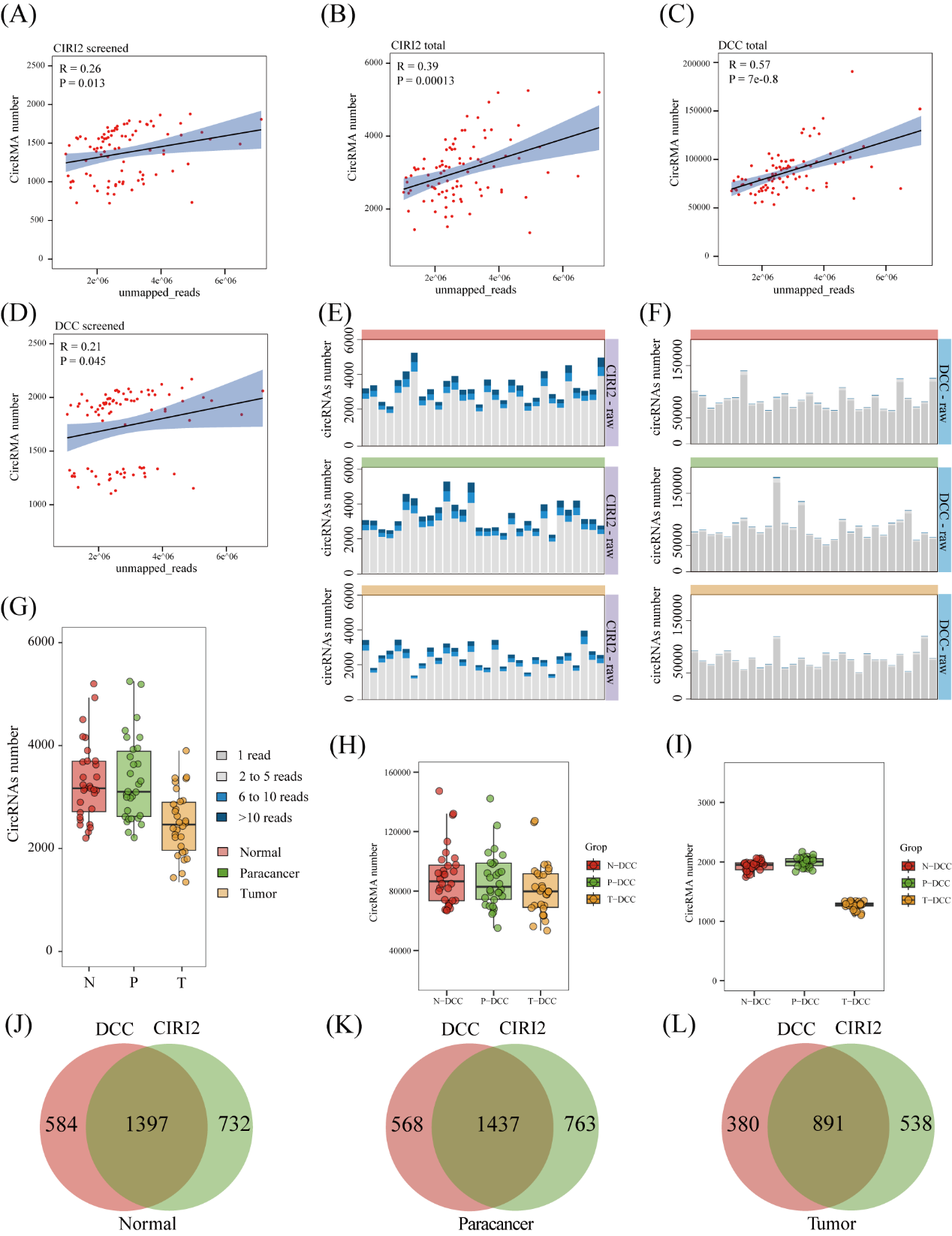


**FIGURE S1** Comprehensive analysis of circRNA detection and distribution across normal, paracancer, and tumor tissues. (A-D) Correlation analysis between unmapped reads and the number of circRNAs detected by each circRNA detection tool. (E, F) Number of circRNAs identified from total RNA sequencing data of normal (N, n=30, red), paracancer (P, n=30, green), and tumor (T, n=30, yellow) tissues using CIRI2 (E) and DCC (F). Each bar represents one patient sample. (G) Boxplot comparing the number of identified circRNAs across different tissue types (N, P, and T). (H, I) Number of identified circRNAs for each patient in each type of tissue. (J-L) Venn diagrams showing the overlap in candidate circRNAs identified by both CIRI2 and DCC tools in normal (J), paracancer (K), and tumor (L) tissues.


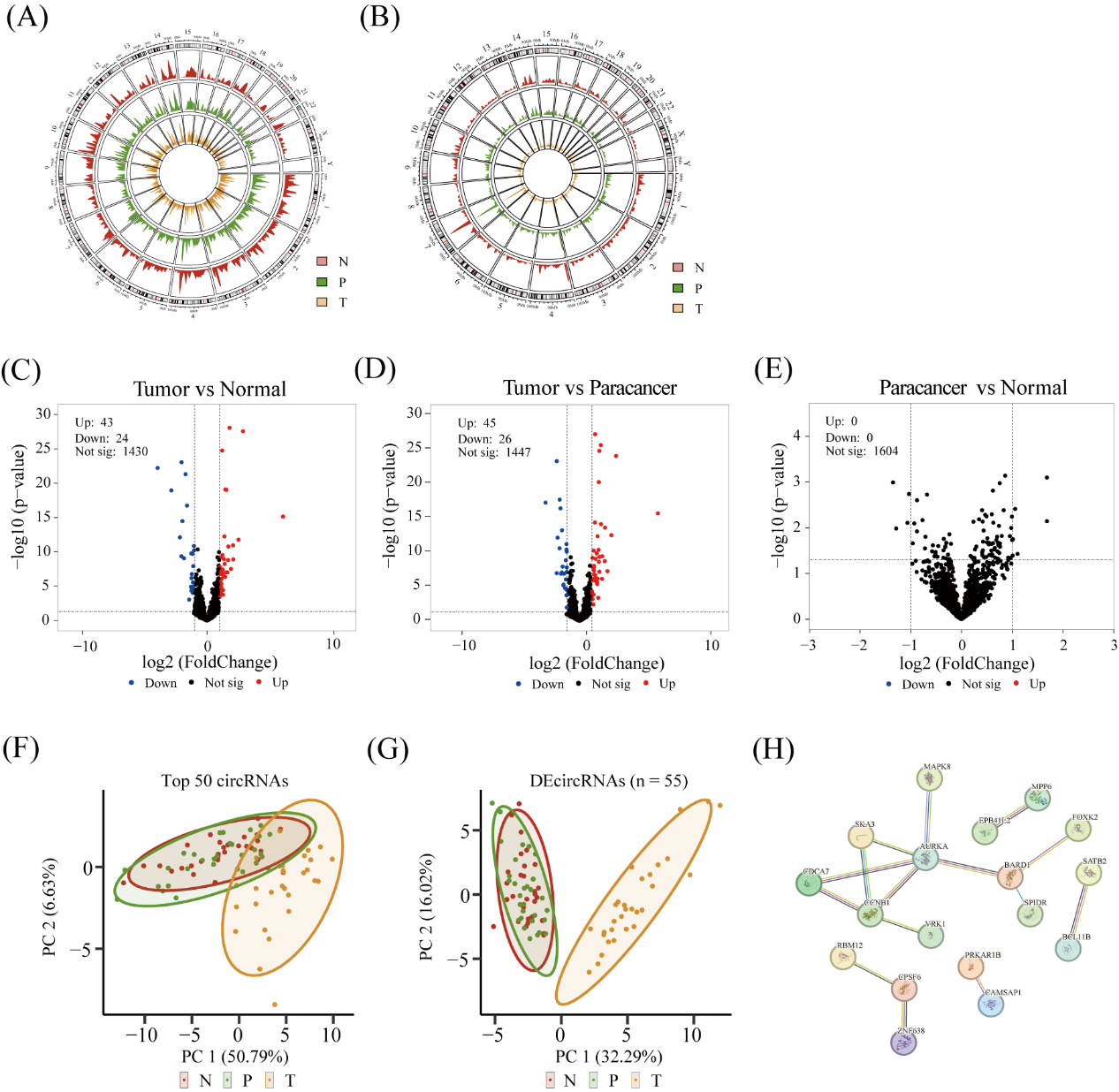


**FIGURE S2** Chromosomal distribution, differential expression, and functional analysis of circRNAs in CRC tissues. (A, B) Chromosomal map of all circRNAs identified using CIRI2 and DCC. (C-E) Volcano plots depicting DEcircRNAs between tissue types: tumor vs. normal (C), tumor vs. paracancer (D), and paracancer vs. normal (E). (F, G) Principal component analysis (PCA) using the 50 most expressed circRNAs and 55 DEcircRNAs. (H) Potential functional relationships between the host genes of the circRNAs.


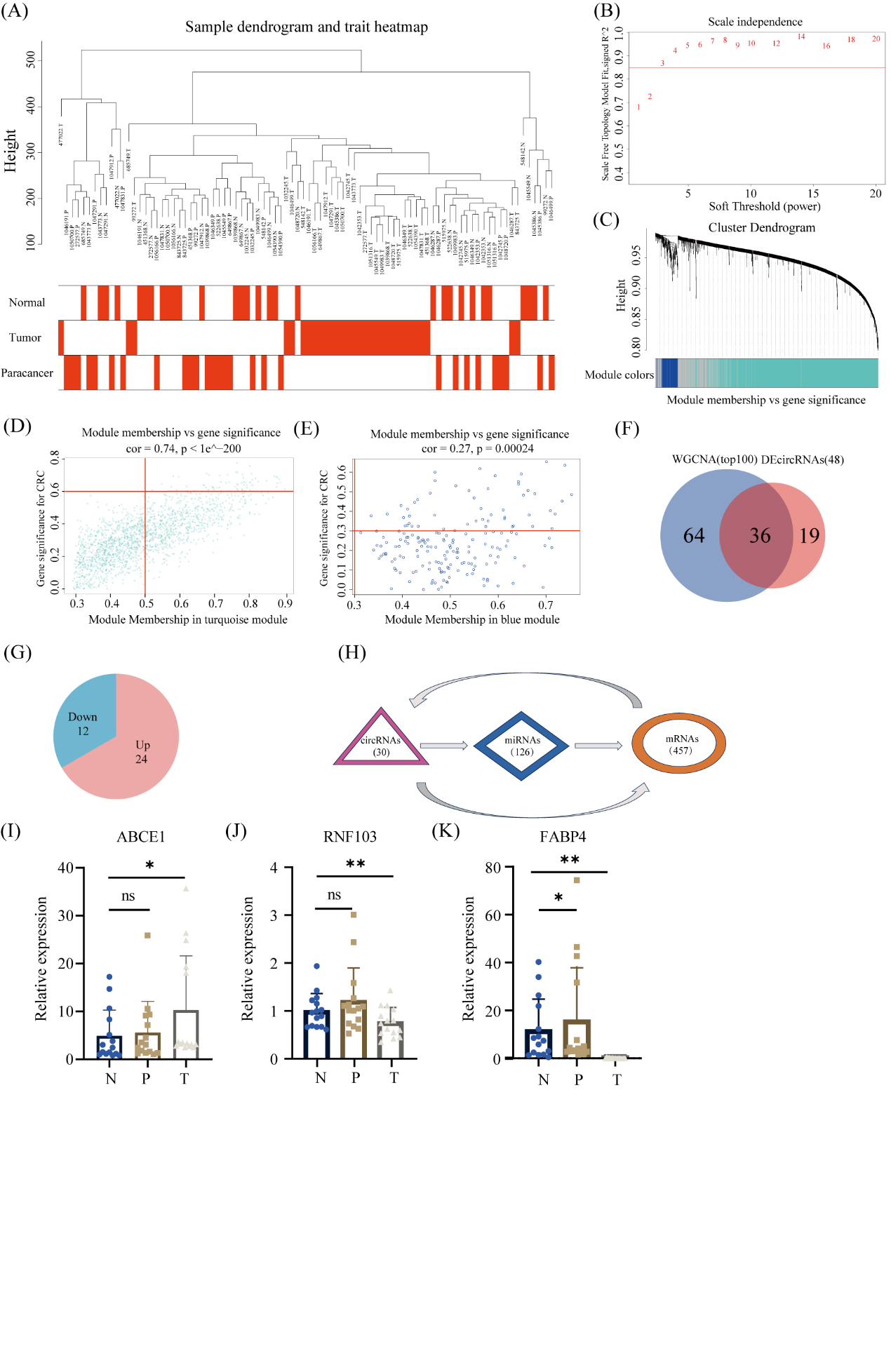


**FIGURE S3** WGCNA of circRNA in CRC tissues. (A) Sample clustering dendrogram for outlier identification. (B) Scale independence and mean connectivity plots for different soft-thresholding powers in each tissue type, with the chosen power (β) marked with a red dot. (C) Gene dendrogram and module assignments before merging, shown separately for each tissue type. (D, E) Scatterplots showing the relationship between module membership (MM) and gene significance (GS) for the turquoise (D) and blue (E) modules in each tissue type. (F) Venn diagram showing the overlap between DEcircRNAs and tumor-specific circRNAs identified by WGCNA. (G) Pie chart displaying the distribution of upregulated and downregulated expression levels among 36 DEcircRNAs. Red represents upregulation, blue represents downregulation. (H) Network representation of the predicted circRNA-miRNA-mRNA regulatory axes, involving 30 circRNAs, 126 miRNAs, and 457 mRNAs. (I-K) Expression profiles of the targeted mRNA in hsa_circ_0001461, hsa-miR-145-5p, and ABCE1 (I); hsa_circ_0019223, hsa-miR-215-3p, and RNF103 (J); hsa_circ_0087960, hsa-miR-30c-1-3p, and FABP4 regulatory axes (K).

**
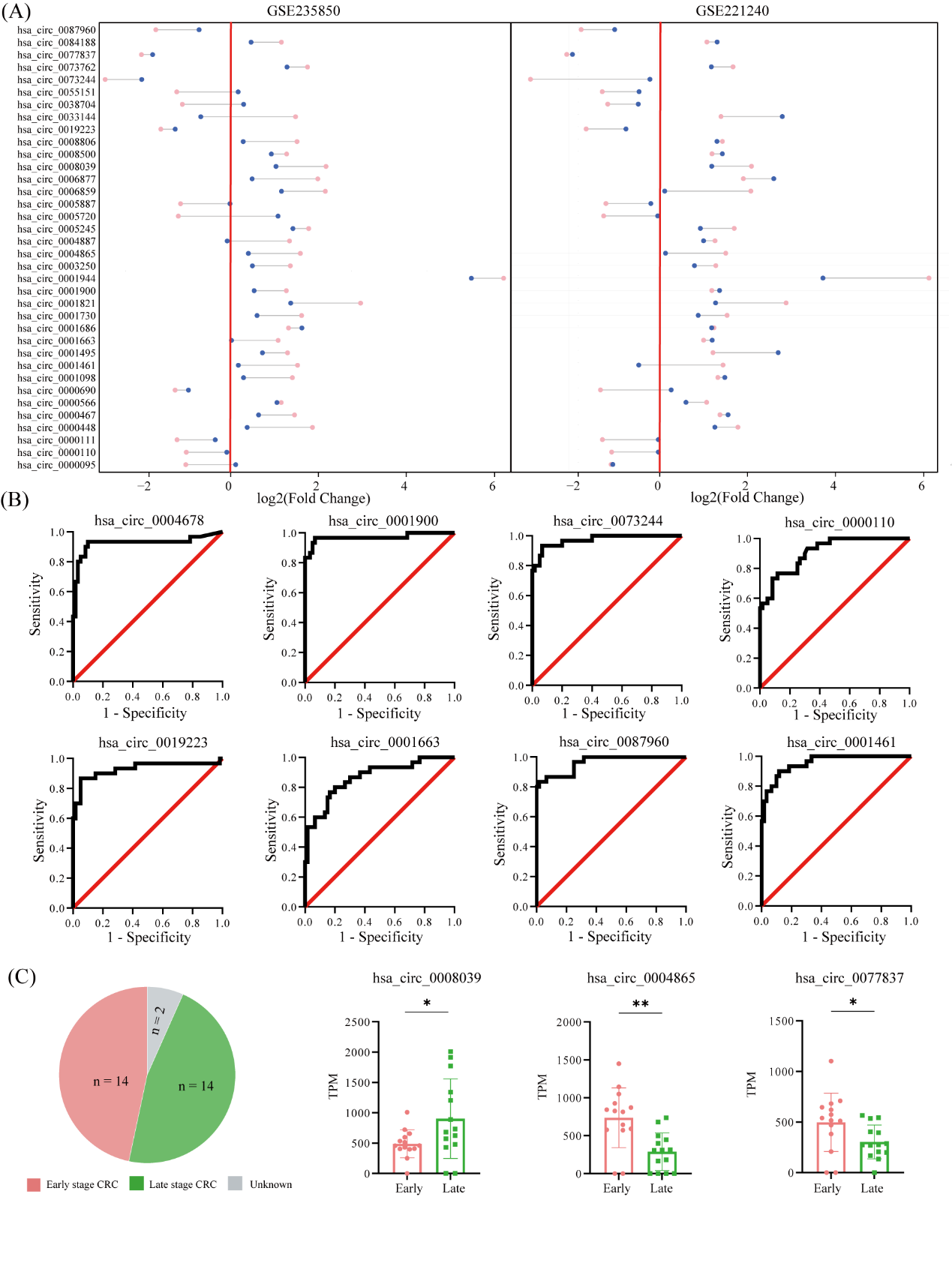
**

**FIGURE S4** Validation and diagnostic potential of DEcircRNAs. (A) Validation of DEcircRNAs using public databases (GSE235850 and GSE221240). (B) ROC curves showing the diagnostic potential of the top 8 DEcircRNAs (based on AUC scores) for distinguishing between tumor and normal tissues. (C) Association of three circRNAs (hsa_circ_0008039, hsa_circ_0004865, hsa_circ_0077837) with early-stage CRC (I and II) compared to late-stage CRC (III and IV) among 28 patients. Significance levels are indicated by *p < 0.05, **p < 0.01. Data are presented as mean ± SEM, n = 28 per group.

**
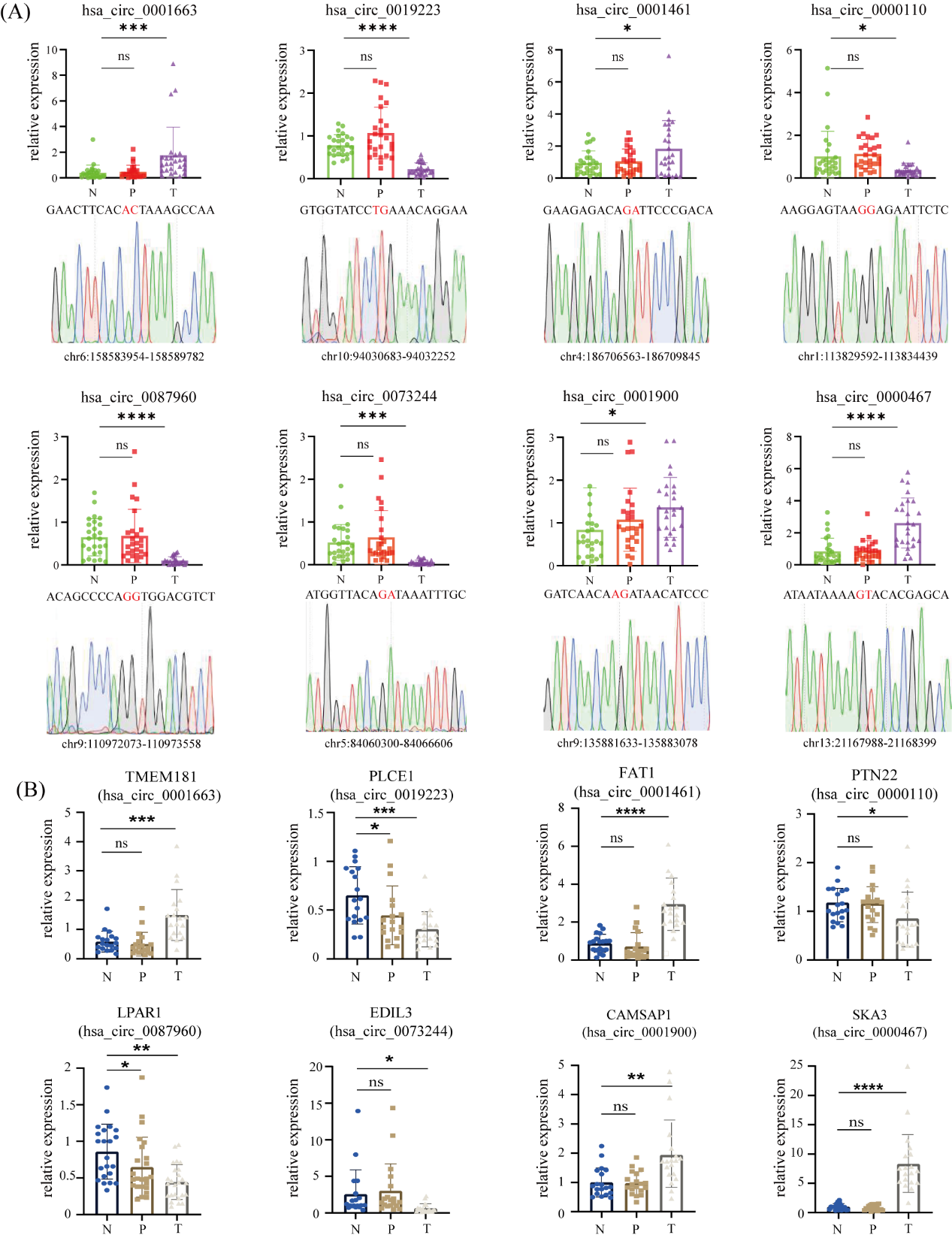
**

**FIGURE S5** Validation of differential expression for selected circRNAs and their host genes in original tissue samples. (A) qRT-PCR validation and sequence chromatograms showing the BSJ of eight DEcircDNAs. (B) Expression profiles of the host genes across normal, paracancer, and tumor tissues. *p < 0.05, **p < 0.01, ***p < 0.001, ****p < 0.0001. Data are presented as mean ± SEM. N, normal tissue; P, paracancer tissue; T, tumor tissue. The bases highlighted in red represent the BSJ regions of the circRNAs.


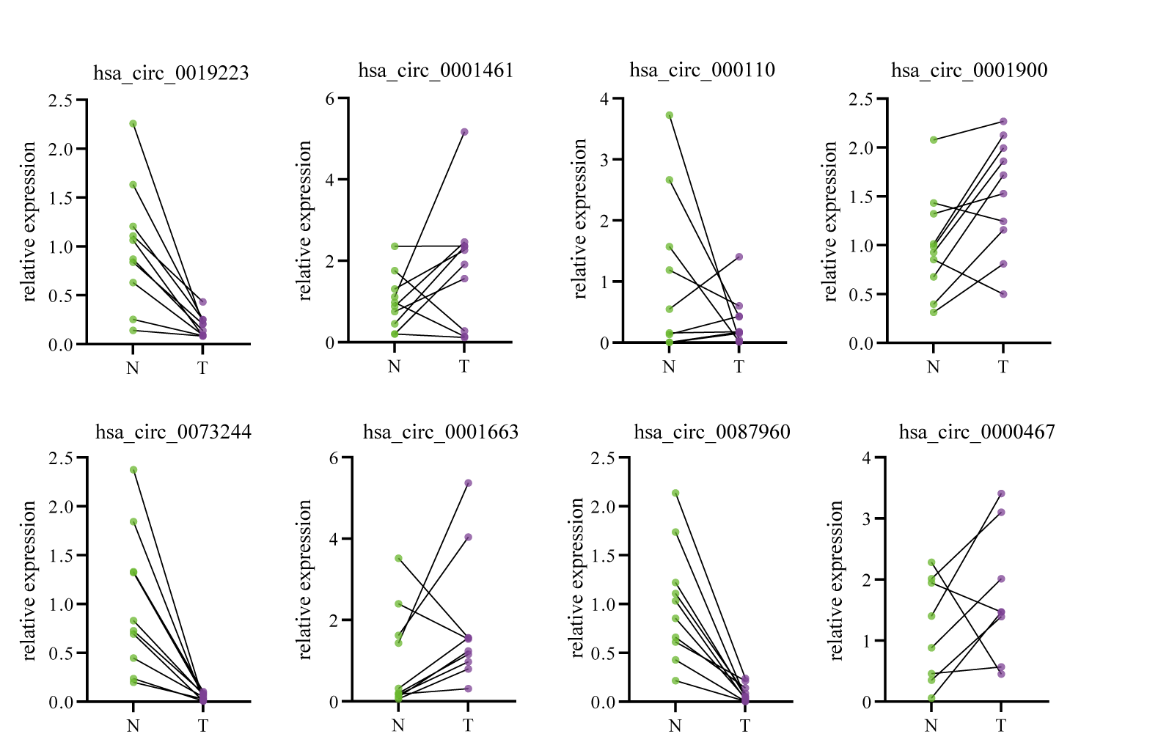


**FIGURE S6** Validation of expression differences for the 8 DEcircRNAs using an additional 10 pairs of CRC tumor-normal tissue samples.


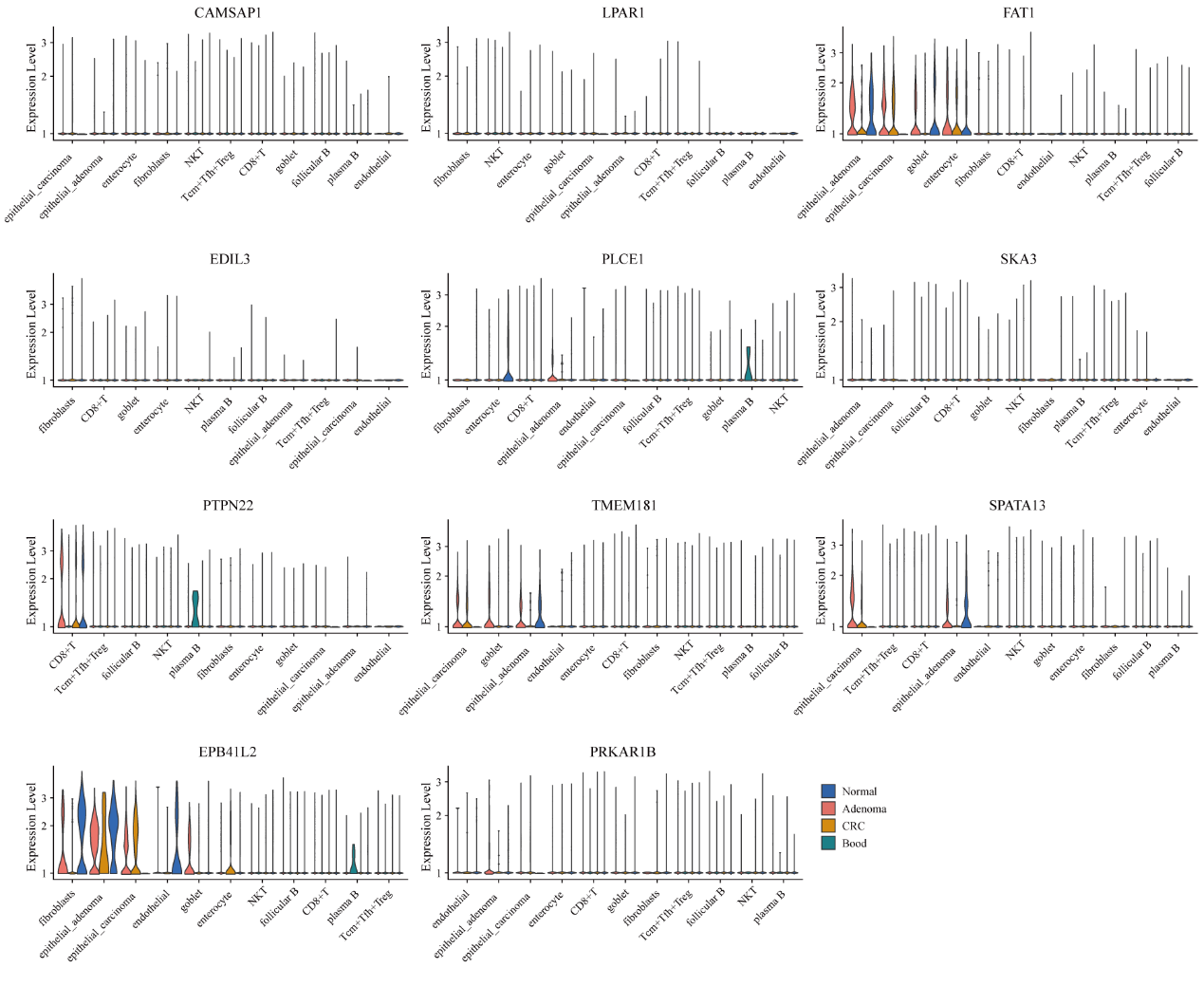


**FIGURE S7** Violin plots illustrating the expression levels of host genes in each cell type among normal tissues, paracancer tissues, tumor tissues, and plasma samples. Blue represents normal tissues, pink represents paracancer tissues, yellow represents tumor tissues, and green represents plasma samples.
