# Supplementary material for: Comprehensive circular RNA profiling provides insight into colorectal cancer pathogenesis and reveals diagnostically relevant biomarkers
Source: Clin Transl Med. 2024 Oct 13;14(10):e70049. doi: 10.1002/ctm2.70049 (PMC11471576; doi:10.1002/ctm2.70049)
Supplement: Supplementary file 2 — Supporting information [file CTM2-14-e70049-s009.docx]

**Supplementary methods**

**Clinical tissue sample collection**

Tissue samples were obtained from 30 CRC patients undergoing surgical resection of malignant tumors at Shenzhen First People's Hospital (Shenzhen, China). For each patient, three distinct tissue types were collected: normal intestinal tissue, paracancer tissue, and tumoral tissue. Immediately after surgery, tissues were meticulously washed with sterile saline and immersed in pre-cooled MACS tissue storage solution (Miltenyi Biotec, 130-100-008). Written informed consent was acquired from all patients, and detailed clinical characteristics are provided in Table S1.

**Whole transcriptome sequencing**

1 ml of RNAiso Plus (Takara, Japan, #9109) was added to tissue samples, which were then homogenized using a tissue grinder. Total RNA was extracted using the phenol-chloroform extraction method. RNA quality and quantity were assessed using the NanoDrop 2000c spectrophotometer (Thermo Fisher Scientific, USA). Subsequently, ribosomal RNA (rRNA) depletion was performed using the Ribo-off rRNA Depletion Kit (Vazyme, China, N406-02). Libraries preparation was conducted by Geneplus Technology Co. Ltd. (Shenzhen, China), and the quality of each library was rigorously evaluated using the Agilent Bioanalyzer 2100 system (Agilent, USA). Finally, sequencing was conducted on the DNBSEQ-T7 platform, generating approximately 12G total reads per library.

**Identification of circRNAs**

Raw reads underwent quality control using FastQC (v0.11.8). Adapter sequences and low-quality reads were removed using cutadapt (v2.10) and trimmomatic (v0.39), generating clean reads for further analysis. Both STAR (v2.7.8) and bwa (v0.7.17) were utilized to align clean reads to the reference human genome (GRCh38) for each sample, producing BAM files. Unmapped reads extracted from BAM files using samtools (v1.7) were subsequently analyzed for circRNA identification using CIRI2 (v2.0.6) and DCC (v0.5.0). Only circRNAs detected in at least half of the patient samples were retained for further investigation.

**Identification of differentially expressed circRNAs (DEcircRNAs)**

DEcircRNAs were identified for each pairwise comparison of tissue groups: normal (N) vs. paracancer (P), normal (N) vs. tumor (T), and paracancer (P) vs. tumor (T). CircRNAs detected in both tissues within each comparison were considered for further analysis and used to generate a circRNA expression read count matrix. DEseq2 software (v4.3.2) was employed to identify DEcircRNAs exhibiting significant changes (|Log2FoldChange| ≥ 1, adjusted p-value < 0.05, and base mean ≥ 3.5) within each group comparison. The top 50 most highly expressed circRNAs along with the identified DEcircRNAs from all three comparisons were included in a principal component analysis (PCA) using the R package ggplot2 (v4.2.3).

**Correlation analysis of DEcircRNAs with corresponding host genes**

The corresponding host genes for each circRNA were extracted from the CIRI2 software output. For each pairwise group comparison (normal vs. paracancer, normal vs. tumor, paracancer vs. tumor), expression read count matrices of mRNAs were generated using featureCounts (v2.22). Subsequently, R package DEseq2 (v4.3.2) was employed to calculate mRNA fold changes within each group. Pearson correlation analysis was then performed to assess the relationship between the Log2FoldChange values of host genes and DEcircRNAs. The STRING database (v12.0) was utilized to construct a protein-protein interaction (PPI) network for the identified host genes.

**Weighted gene co-expression network analysis (WGCNA)**

WGCNA was applied to identify groups of co-regulated circRNAs associated with CRC progression. The input matrix contained the expression profiles of 2,390 circRNAs identified across the three tissue types (Table S3). The analysis was performed using the WGCNA R package (v4.3.2) and involved three key steps: 1. Outlier sample removal: To ensure robust network construction, clustering analysis was performed on the expression profiles of all 90 tissue samples. Samples identified as outliers based on clustering patterns were excluded, resulting in a final dataset of 88 tissue samples for further analysis. 2. Soft threshold selection: To define an appropriate level of network interconnectedness, the optimal soft threshold was determined using the “pickSoftThreshold” function, ensuring a correlation coefficient greater than 0.85. Subsequently, a β value of 3 was applied to convert the similarity matrix into an adjacency matrix, which represents the network connections between circRNAs. 3. Module identification: Based on the topological overlap matrix, co-expressed circRNAs were clustered into modules using a hierarchical clustering approach. Modules exhibiting significant associations with disease groups were identified and selected for further investigation.

**Construction of circRNAs-miRNAs-mRNAs regulatory network**

Miranda (v3.3a) was used to predict miRNA binding sites on each DEcircRNAs, high-scoring alignments were selected for further analysis (energy ≤ -7, score ≥ 150). We obtained the CRC miRNA expression matrix from the dbDEMC database (https://www.biosino.org/dbDEMC/index) and filtered for miRNAs exhibiting significant expression changes during disease progression (|Log2(FoldChange)| ≥ 0.5, adjusted p-value < 0.05). We then intersected these DEmiRNAs with those predicted by miRanda for each DEcircRNA. Finally, miRNAs with expression trends consistent with their corresponding circRNAs were excluded. For each remaining miRNA, the top 5 scoring mRNA targets were retrieved from TargetScanHuman (v8.0), again filtering for mRNAs with significant expression changes during disease progression (|Log2(Fold Change)| ≥ 0.5, adjusted p-value < 0.05) and discarding those with expression trends mirroring the miRNAs. Cytoscape (v3.10.1) software was then utilized to visualize the final circRNAs-miRNAs-mRNAs regulatory network.

**Functional enrichment analysis**

We performed functional enrichment analysis on the targeted mRNAs using the R package clusterProfiler (v4.0.0). This analysis included Gene Ontology (GO) for biological processes, Kyoto Encyclopedia of Genes and Genomes (KEGG) for signaling pathways, and Gene Set Enrichment Analysis (GSEA) for coordinated gene expression changes. In the GO and KEGG analysis, pathways with statistically significant P-values (< 0.1 and <0.01, respectively) were selected and visualized as bar graphs for clarity. GSEA employed the built-in functions of clusterProfiler to generate ridge plots, with four cancer-related pathways chosen for individual presentation based on their relevance and enrichment scores.

**Real-time quantitative reverse transcription PCR (qRT-PCR)**

qRT-PCR was conducted using the remaining tissue samples. In general, total RNA was extracted from tissues using RNAiso Plus (Takara, Japan, #9108) and its quality was assessed by measuring the OD260/280 ratio (1.8-2.0). High-quality RNA was reverse transcribed into cDNA using M-MuLV reverse transcriptase (FAPON, China, MD028). Quantitative real-time PCR was performed using SYBR Green master mix (Applied Biosystems, USA, A25741) and specific back-splice junction (BSJ) divergent primers designed for each circRNA or primers for mRNAs (primer sequences provided in Table S7). The relative expression levels of circRNAs were normalized using β-actin, and gene expression was calculated using the 2^-ΔΔCt^ method.

**Validation using GEO datasets**

Two independent expression matrices of CRC circRNAs were obtained from the GEO database (GSE221240 and GSE235850). For each circRNA in both datasets, the normalized Log2FoldChange was calculated using the R package DEseq2 (v4.2.2). Subsequently, Receiver Operating Characteristic (ROC) curves were generated using GraphPad Prism 9.0 software to assess the discriminative ability of the DEcircRNAs in differentiating tumors from normal tissues.

**Single cell RNA sequencing (scRNA-seq) data analysis**

The scRNA-seq data from 13 CRC samples was retrieved from the GEO database (GSE161277). Data analysis was performed using the Seurat R package (v4.2.3) after applying strict quality control filters. Each gene had to be expressed in at least 3 cells, and each cell had to express at least 250 genes. Additionally, gene expression levels ranged from 100 to 7500 with mitochondrial content below 35% and UMI exceeding 1000. This rigorous filtering resulted in 43,851 high-quality cells for further analysis. Prior to clustering, data was normalized using the “NormalizeData” function and variable genes were identified based on variance stabilization transformation (vst) using the “FindVariableFeatures” function. All genes were then scaled via the “ScaleData” function. Principal component analysis (PCA) was performed on the scaled data, and the optimal dimensionality was determined to be 30 dimensions using the “JackStraw” and “ElbowPlot” functions. Subsequently, cell clustering was conducted with “FindNeighbors” and “FindClusters” functions (Resolution = 0.4), resulting in 21 distinct clusters. Finally, human cell marker genes from <http://biocc.hrbmu.edu.cn/CellMarker/> were used to annotate cell types.

**Cell culture and transfection**

Three CRC cell lines (HCT116, LoVo, SW480) and the normal colonic cell line (NCM460) were cultured in high-glucose Dulbecco’s modified Eagle medium (DMEM) supplemented with 10% fetal bovine serum (FBS, Gibco) at 37°C with 5% CO2. Small interfering RNAs for hsa_circ_0019223, hsa_circ_0001461, and hsa_circ_0087960 siRNA, as well as their corresponding control oligonucleotides, were purchased from GenePharma (GenePharma Corporation, Shanghai, China). Transfection was performed using RNAimax reagent (Thermo Fisher Scientific Inc. Massachusetts, USA) according to the manufacturer’s instructions. The siRNA sequences are provided in Table S8.

**Proliferation Assay**

The proliferation ability of CRC cells was assessed using the Cell-Light EdU Apollo567 In Vitro Flow Cytometry Kit (RIBOBIO, Guangzhou, China). The procedure was as follows: siRNA-treated CRC cells were seeded into 48-well plates and cultured until 60-70% confluence. The cells were then incubated in DMEM medium containing 20 μM EdU for 2 hours, followed by fixation with 4% paraformaldehyde at room temperature for 30 minutes. After fixation, the cells were permeabilized with 0.5% Triton X-100 for 10 minutes and then stained with 1× Apollo reaction cocktail for 1 hour, followed by Hoechst staining for 10 minutes. Images were captured and analyzed using the Lionheart FX Automated Live Cell Imager (BioTek, USA).
